# Association of cetylated fatty acid treatment with physical therapy improves athletic pubalgia symptoms in professional roller hockey players

**DOI:** 10.1016/j.heliyon.2020.e04526

**Published:** 2020-07-28

**Authors:** Enrico Pampaloni, Elena Pera, Duilio Maggi, Riccardo Lucchinelli, Dante Chiappino, Andrea Costa, Veronica Venturini, Germano Tarantino

**Affiliations:** a Physioterapy Center, Physio Point, Lucca, Italy; b Alesco Srl, Pisa, Italy; c USL 12, Versilia, Italy; d Diagnostic Imaging OU, Massa Hopsital, Massa, Italy; e Pharmanutra SpA, Pisa, Italy

**Keywords:** Musculoskeletal system, Medical imaging, Clinical research, Health profession, Diagnostics, Athletic pubalgia, Cetlyated fatty acids, Physical therapy, Sports hernia, Strength test, Ultrasound

## Abstract

**Background:**

Athletic pubalgia (AP), a frequent problem among professional roller hockey players (PRHPs), consists of lower abdominal and groin pain, without the presence of true hernia.

**Aims:**

We assessed cetylated fatty acids (CFAs) in association with conservative therapy for treatment of AP in PRHPs.

**Methods:**

Ultrasound examination was performed before and after treatment. Strength tests were performed and AP-related pain was measured during the treatment period.

**Findings:**

Nine of 10 enrolled PRHPs completed a 12-week treatment with CFAs in association with conservative therapy, consisting of manual therapy, diathermy or ultrasonography. Initial ultrasound examination showed AP signs in 7 (70%) of 10 PRHPs. After 12 weeks of therapy, these signs could only be detected by ultrasound in 2 (22.2%) of 9 PRHPs. An increase in muscle strength (already after first week of treatment) and a reduction of AP-related pain were also observed during the treatment.

**Conclusion:**

The association of CFA treatment with a conservative rehabilitation therapy improves muscle strength and pain and may accelerate recovery from AP.

## 1 Introduction

Athletic pubalgia (AP) is a frequent problem in professional athletes and is characterized by a chronic lower abdominal and groin pain without the presence of a true hernia (Cohen et al., 2016). AP may be caused by an injury of different structures of the pubic aponeurosis (Malycha and Lovell, 1992; Polglase et al., 1991; Taylor et al., 1991), and it is manly present among athletes who are practicing sports that require rapid changes in direction, frequent side-to-side motions and quick acceleration such as soccer, hockey, baseball and fencing (Cavalli et al., 2014; Elattar et al., 2016; Omar et al., 2008; Renstrom and Peterson, 1980). The presence of chronic AP may result in debilitating pain and loss of playing time, and may lead to an early termination of the athlete's career (Omar et al., 2008). A clear diagnosis of AP can be sometimes difficult due to the anatomic complexity of the area and to other, even concomitant, pathological conditions that may results in similar clinical signs and symptoms (Ekberg et al., 1988; Elattar et al., 2016; Ellsworth et al., 2014; Omar et al., 2008). Therefore, diagnosis of AP is challenging, also for athletic trainers, team physicians and physiotherapists. Treatment of AP may consist in conservative or surgical treatment, even if the latter is limited to patients that, after a nonsurgical approach, continue to have pain. Indeed, the first-line approach consists in rest, anti-inflammatory drugs and, most importantly, rehabilitation using physical therapy (Cohen et al., 2016; Elattar et al., 2016; Ellsworth et al., 2014).

Cetylated fatty acids (CFAs), which are fatty acids esterified with cetyl alcohol have shown to have a role in protecting synovial membranes and stabilizing cell membranes, allowing for normal flexibility and mobility of joints, and resulting in a reduction of pain and increased fluid at joints level, which contributes to their normal lubrication (Diehl and May 1994; Hesslink et al., 2002). In professional sport, treatment with CFAs might be beneficial in improving the elasticity and resistance of the synovial membranes through a lubrication effect, and in decreasing pain due to high physical activity or following an injury. Since professional roller hockey players (PRHPs) present injuries at groin level (Varlotta et al., 2000), and they are often affected by chronic AP, CFAs may be useful as adjuvant to a conservative therapy in their treatment. In this study, we evaluated the effect of topical CFA application on muscular function and AP-related pain.

## 2 Results

### 2.1 Baseline characteristics of the PRHPs

The initial ultrasound revealed that 7 (70%) of the 10 PRHPs had insertional calcifications, and of these 7, 4 presented them on only one limb and 3 bilaterally. Moreover, most of these 7 PRHPs also showed liquid deposits and/or fibrosis around the adductor muscles. The 3 PRHPs who did not show major signs of AP at these initial examinations, started and continued on manual therapy for the entire treatment period, unless injury or muscle fatigue occurred, while the 7 PRHPs who showed major signs of AP were started on diathermy or ultrasonography for the first 2–3 weeks and then continued on manual massage, unless injury or muscle fatigue occurred. One PRHP withdrew from the study after 1 week for reasons not linked to the study.

### 2.2 Strength and pain measurements

**Figure 1:**
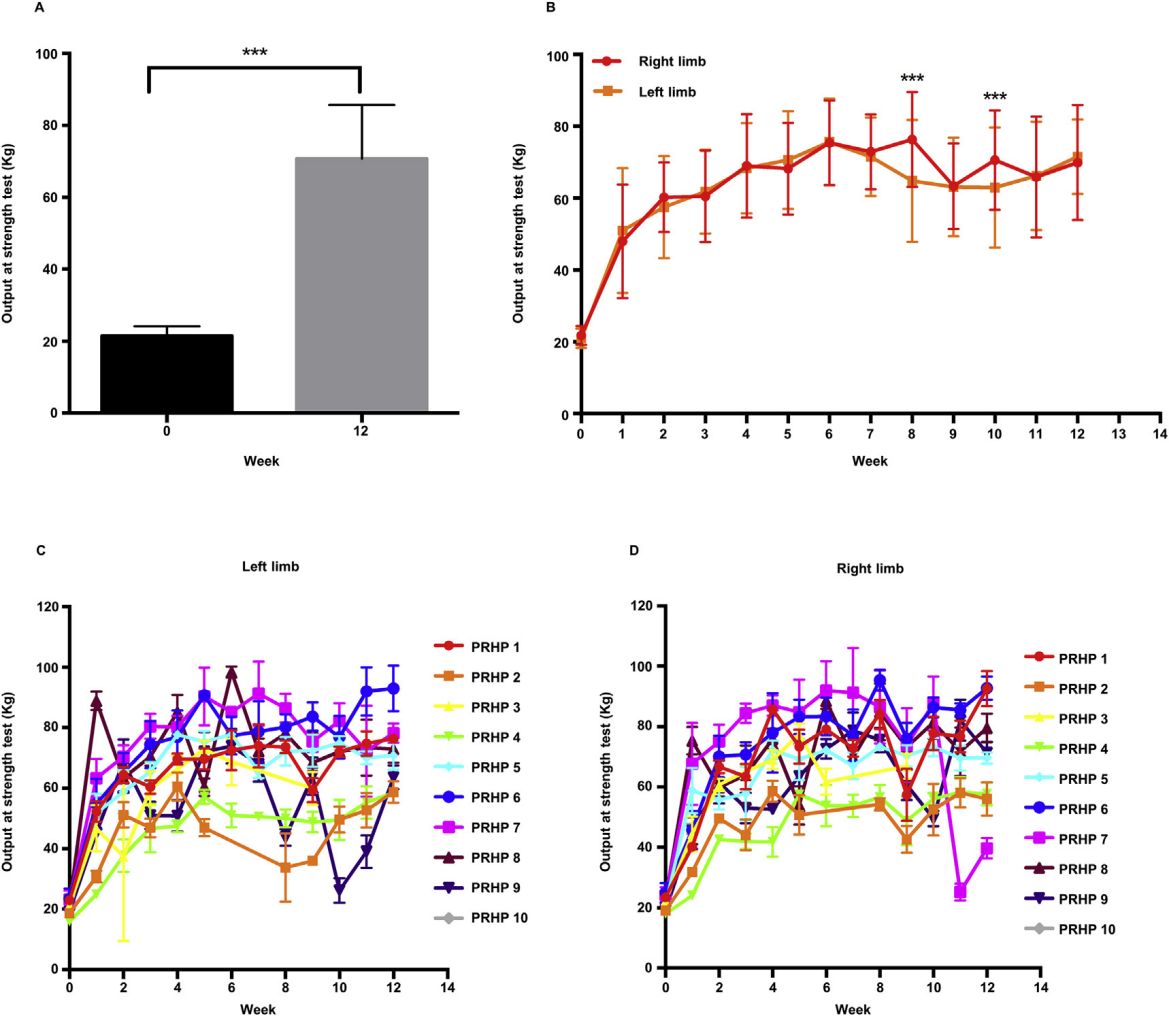
Results of the strength test. **A)** Overall results from the strength test, pooled data from both limbs shows a significant difference between week (W)0 and W12. Unpaired sample *t* test, *p* ≤ 0.001. **B)** Overall results for each limb during the period studied. Two-way ANOVA with Sidak post-analysis test, *p* ≤ 0.001. **C)** Average pain measurement of each PRHP for the left limb. **D)** Average performance of each PRHP for the right limb. PRHP, professional roller hockey player; kg, kilogram.

The results of the strength tests showed a significant increase between the baseline value and the values recorded after 12 weeks of treatment, with a fold-increase of about 3.22 between week (W)0 and W12 (Figure 1A). The measurements were comparable between the two limbs (Figure 1B), and combining them, the overall baseline value was 21.59 ± 2.69 kilograms (kg), which increased to 69.45 ± 15.61 kg (*p* < 0.001) after 12 weeks of treatment. Interestingly, the major and significant increase in strength was observed after the first week of treatment, and was approximately 2.25-fold (to 48.60 ± 15.61 kg) compared to baseline (Figure 1B). A slight decrease in strength was recorded between W8 and W10 (Figure 1B, C and D), which could be attributed to injury and muscle fatigue reported by 3 PRHPs during that period (data not shown).

**Figure 2:**
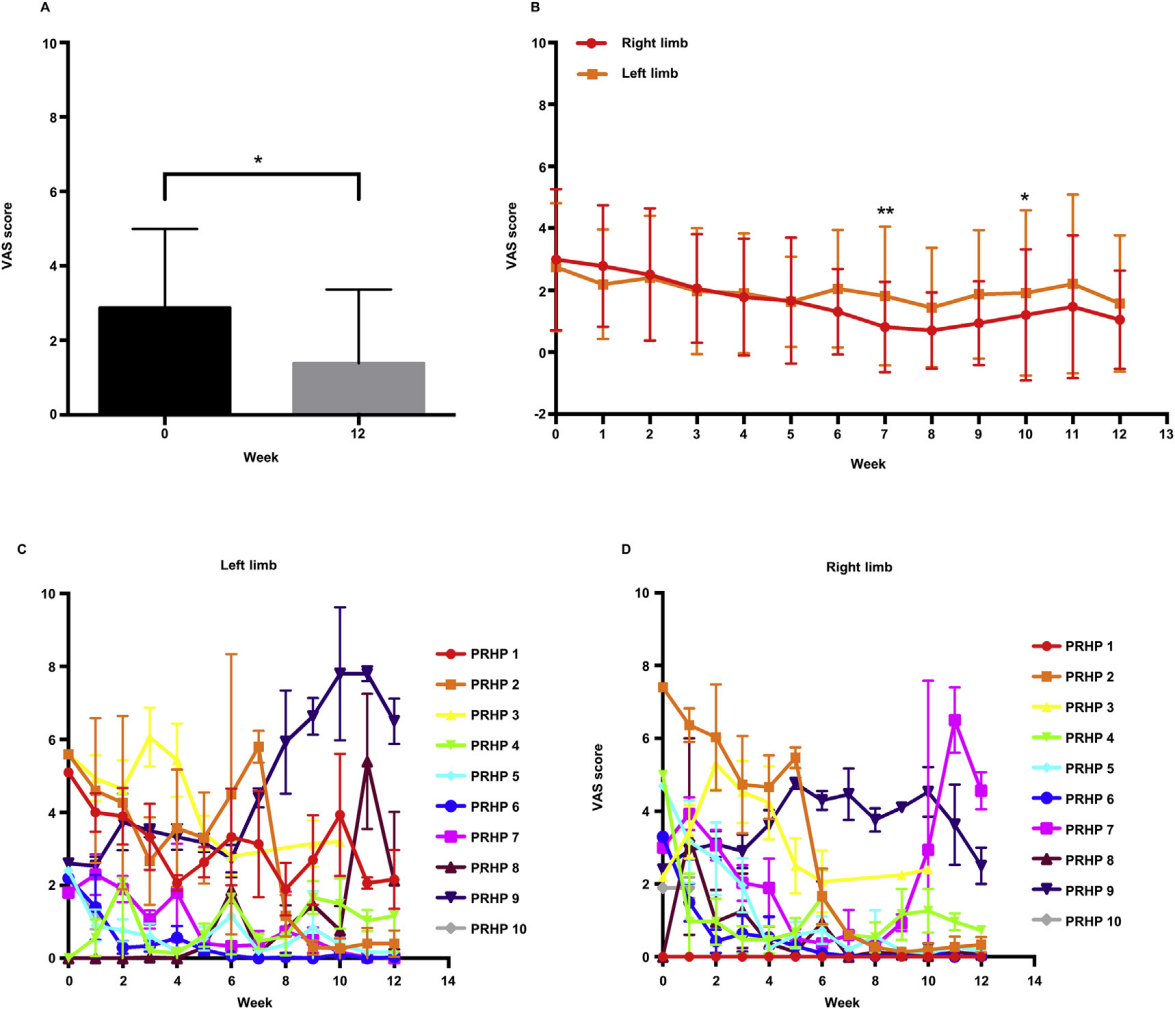
Results from pain measurement using the VAS score. **A)** Overall results from the pain measurement, pooled data from both limbs shows a significant difference between week (W)0 and W12. Unpaired sample *t* test, *p* ≤ 0.05. **B)** Overall results for each limb during the period studied. Two-way ANOVA with Sidak post-analysis test, *p* ≤ 0.001. **C)** Average performance of each PRHP for the left limb. **D)** Average performance of each PRHP for the right limb. PRHP, professional roller hockey player; VAS, visual analogue scale.

AP-related pain, measured using visual analog scale (VAS) score, showed a significant decrease in pain at the end of the 12 weeks of treatment with a 2-fold decrease compared to baseline (Figure 2A). This result was statistically significant despite injuries and muscle fatigue reported during the period taken into consideration. The VAS scores for the two limbs were statistically different at W7 and W9 (Figure 2B, C and D), which was the period when most of the PRHPs reported some physical issues, mainly for the left limb (data not shown).

### 2.3 Ultrasound results

**Figure 3:**
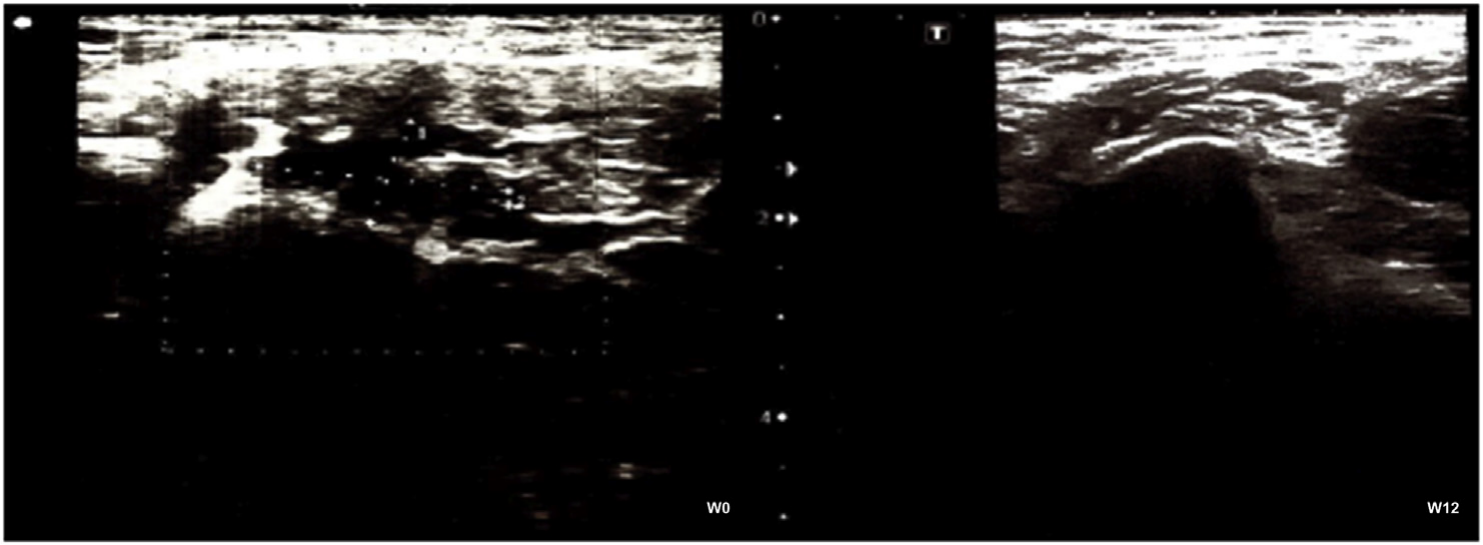
Ultrasound examination. Representative ultrasound examination at week (W)0 and at W12 of a professional roller hockey player who presented calcifications and liquid deposit before beginning the treatment. At the end of the treatment, calcifications and liquid deposit were substantially reduced.

Before the treatment, the ultrasound of most PRHPs showed calcifications, liquid deposit and lesions (Figure 3), as expected in PRHPs. Although the treatment period corresponded to the peak of the season, the ultrasound examinations after 12 weeks of treatment showed a decrease in the number of lesions, calcifications and liquid deposits (Figure 3), resulting in an improvement in terms of tendon and muscle status. At W12, only 2 (22.2%) PRHPs presented calcifications, which were however reduced compared to the initial examination. No liquid deposits or fibrosis were detected.

### 2.4 Case study

**Figure 4:**
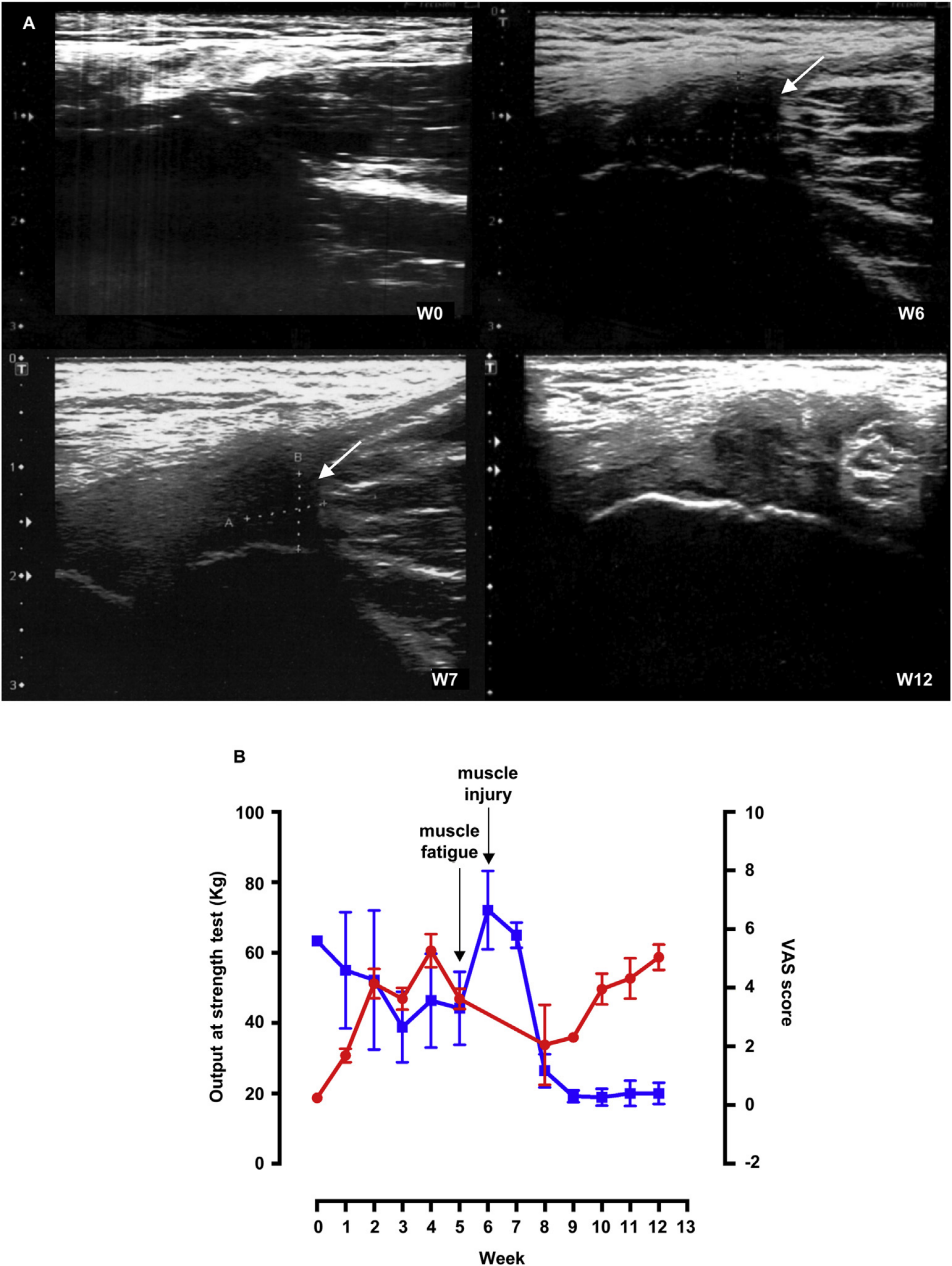
Case-report of traumatic injury. **A)** Ultrasound examinations at week (W)0, at the time of the injury (W6), one week after the injury (W7) and at the end of the treatment (W12). At W6 an edema is visible, which is already reduced after 1 week and is barely visible at W12 with no residual sign of injury. **B)** Strength and pain evaluations of the professional roller hockey player who had a traumatic injury. kg, kilogram; VAS, visual analogue scale.

One PRHP included in the study reported a traumatic injury during the study period. At the initial examination at W0, the PRHP presented a small liquid deposit at the right adductor longus with calcification, while none was reported for the left adductor (Figure 4A, upper left), and thus, he started on manual therapy and CFA topical application. During the first 5 weeks, he showed a significant increase in strength and a decrease in the VAS score (Figure 4B). At W5, he suffered from muscle fatigue, followed at W6 by an injury with an edema of 12 × 11 × 8 mm at the left adductor longus with muscle fibers rupture (Figure 4A, upper right). The injury was caused at the end of the match by a fast change in direction from a standing position followed by a quick restart. Thus, after the injury, the strength test was not performed, and manual therapy was replaced with diathermy in association with CFA treatment. One week later, another ultrasound examination was performed, which revealed a dimensionally reduced edema (8 × 7 × 8 mm) in state of re-organization and in absence of residual liquid deposit or pathological calcifications (Figure 4A, lower left), which was also confirmed by the subsequent ultrasound at W8. Accordingly, after only two weeks, the PRHP showed a significant reduction in pain, with a VAS score lower than the one recorded before the injury (Figure 4B), and due to this fast recovery, he could return to play. Moreover, despite the injury, the muscle strength of the PRHP was regained by W12, reaching a similar level to the one recorded before the injury. Finally, at W12 the ultrasound showed no residual signs of injury and a barely visible edema (Figure 4A, lower right).

## 3 Discussion

The present study aimed to evaluate a possible protocol for the nonsurgical treatment of AP. Overall, the results show that combining CFA treatment and physical rehabilitation, either by using manual therapy or indirect therapy, improves chronic status and helps to accelerate recovery from AP and AP-related injuries. Sports such as roller hockey involve frequent pivoting and cutting, and PRHPs may present high muscle fatigue at adductor level and they can often suffer from injuries at these muscles with possible AP onset (Silvis et al., 2011), which may overlap with the beginning of competitive engagement at professional level.

CFAs are esterified fatty acids that are normally present in the synovial membrane phospholipids, and are characterized by a rapid absorption following topical administration. CFAs can easily permeate the skin owing to a passive and simple chemical-physical gradient favored by the lipidic nature of cell membranes (Sjovall et al., 2018). Therefore, CFAs can be adsorbed at joint level and reach the synovial membrane, and can help to reduce joint pain, leading to an improvement in joint mobility. The use of CFAs in association with therapeutic massage is known to improve and accelerate the rehabilitation period compared to manual therapy alone (Sharan et al., 2011). The CFA products used in this study (Cetilar®, PharmaNutra SpA, Italy) have previously been shown to be effective in the topical treatment of knee osteoarthritis-related pain (Ariani et al. 2016, 2017, 2018).

AP is a chronic condition in athletes that can require up to 6 months of recovery before being able to return to sports (Elattar et al., 2016). Several studies report a significant improvement after 6–8 weeks of physical therapy (Kachingwe and Grech, 2008; Woodward et al., 2012), which may allow the athlete to return to play. Indeed, most of these therapies involve a period of absence from professional activity, during which the physical therapy was performed (Cohen et al., 2016; Elattar et al., 2016; Ellsworth et al., 2014; Paksoy and Sekmen, 2016). In the present study, where conservative therapy was complemented by the use of CFAs, interruption of the professional activity was not needed, unless a traumatic injury occurred, and thus, the AP symptoms were treated during the season activity. Moreover, the study period chosen coincided with the peak of the season, when PRHPs were under a heavy workload, since they were competing in both the Italian A-series tournament and the European championship. Thus, an average of 2–3 matches per week were played during the study period.

The efficacy of the protocol applied in the study was also demonstrated by the fast recovery reported by one of the PRHPs, who suffered from a traumatic injury. The PRHP was able to return to professional activity after only two weeks from AP-related traumatic injury. In addition, the association of CFAs and diathermy appeared to be efficacious in yielding a fast recovery, as also confirmed by the ultrasound examinations. This is in line with previous findings, which show that the association of CFA topical application and a personalized diathermy program helps to reduce pain and to increase functional activity after sport injury (Biondi and Crispino, 2011). Furthermore, the condition of the PRHP appeared to be significantly improved after only 1 week of treatment, as shown by the strength test and VAS score, which continued to improve for the rest of the treatment period. These results were also confirmed by final ultrasound examinations, which despite injuries and muscle fatigue, showed an overall improvement of AP symptoms for all PRHPs.

The most important limitation of this study was the absence of a control group. Even though the sample size of our study was small, most (9 of 10) PRHPs completed the study up to the last assessment. Further studies will be needed to confirm these preliminary findings.

## 4 Conclusions

This study shows that the association of CFA topical application with a conservative therapy may accelerate recovery from AP by improving muscle function and reducing pain compared to conservative AP treatment alone, which is known to require a longer time and absence from sport activity (Cohen et al., 2016; Elattar et al., 2016; Ellsworth et al., 2014; Kachingwe and Grech, 2008; Paksoy and Sekmen, 2016; Woodward et al., 2012).

## 5 Methods

### 5.1 Study design and participants

In this open-labeled study, 10 PRHPs (Table 1) were enrolled during the A-league season. The PRHPs were not using other topical treatment in the pubic area or oral pain reduction therapy prior to the beginning of the study and did not have any ongoing infectious diseases. PRHPs involved in the study started agonistic activity around the age of 4 years and they all started to have AP at the beginning of their professional activity, around the age of 17 years. Before study start, verbal informed consent was obtained from all participants. In addition, the participant involved in the case study consented to publish his individual details. The study was conducted in accordance with the Declaration of Helsinki and the principles of Good Clinical Practice. Owing to its design and population, the present study did not require approval of an ethics committee.

**Table 1:** Baseline characteristics of the 10 professional roller hockey players.

Characteristics	Values
Sex	Male
Age (years ± standard deviation)	31.3 ± 7.0
Body Mass Index (average)	24.3
Age at the beginning of agonistic activity (years ± standard deviation)	4.3 ± 1.3
Age at the beginning of professional activity (years ± standard deviation)	17.4 ± 2.4
Years of professional activity (± standard deviation)	13.9 ± 8.9

### 5.2 Procedures

The treatment consisted in a topical application of CFA (Cetilar®, PharmaNutra SpA, Italy) at the pelvic area on both limbs for 12 weeks in combination with either myofascial massage therapy associated with proprioceptive neuromuscular facilitation technique (Hindle et al., 2012), indirect therapy using diathermy (Dottor Tecar AVX, Mactronic) or ultrasonography (Sonokiné 1Mhz, Elettronica Pagani).

Cetilar® is a commercially available topical product containing a patented formulation of CFAs, from vegetable oils, which helps to reduce pain and an increase joint mobility (Ariani et al. 2016, 2017, 2018). The product is available as cream (Cetilar® Crema) and as patch (Cetilar® Patch), and both forms were used during the study. Every second weekday, CFA cream was associated with either direct (manual) or indirect (instrumental) therapy, while the CFA patch was used every other weekday, with no direct or indirect therapy during Saturday and Sunday, when usually matches took place (Table 2).

**Table 2:** CFA treatment scheme.

Monday	Tuesday	Wednesday	Thursday	Friday	Saturday	Sunday
CFA cream + therapy	CFA patch	CFA cream + therapy	CFA patch	CFA cream + therapy	No CFA, No therapy	No CFA, No therapy

CFA, cetylated fatty acids.

The type of therapy chosen was based upon initial ultrasound examination of the PRHP. Myofascial massage and proprioceptive neuromuscular facilitation therapy were mainly performed on PRHPs who did not show presence of large liquid deposits, calcification or fibrosis, and in this case, 5 ml of CFA cream was used on each limb as massage adjuvant. Diathermy or ultrasonography was instead preferred either for PRHPs who showed signs of AP upon the first examination or as an alternative to manual therapy in case of injury or muscle fatigue. For these therapies, 5 ml of CFA cream was used on each limb. For diathermy, CFA cream was applied in a 1:1 ratio with the conducting gel necessary for diathermy, while for ultrasonography, the CFA cream:conducting gel ratio was 0.5–0.75:1. In both cases, the treatment was performed until the CFA cream was completely absorbed.

At the beginning (W0) and at the end of treatment (W12) an ultrasound examination of the pelvic-ischiatic area was carried out bilaterally to evaluate the status of tendons and muscles as well as the eventual presence of lesions and calcifications and liquid deposits before and after the treatment. More precisely, the detectable structure status of tendon and muscle thickness considered in the study and any differences between the left and right limb were recorded.

An initial assessment of PRHPs was carried out at W0 to evaluate pain, muscle strength and recent/past injuries for both limbs. Muscle strength was evaluated using a digital dynamometer (FK1K, Sauter). The FK1K dynamometer has a range of 1000 N, a resolution of 0.5 N, an accuracy of ± 0.5% and a peak-hold function. The dynamometer was regularly calibrated. Three repeats were performed for each test at intervals of 15 s and the duration of each measurement ranged from 3-5 s. Results are reported in kg.

**Figure 5:**
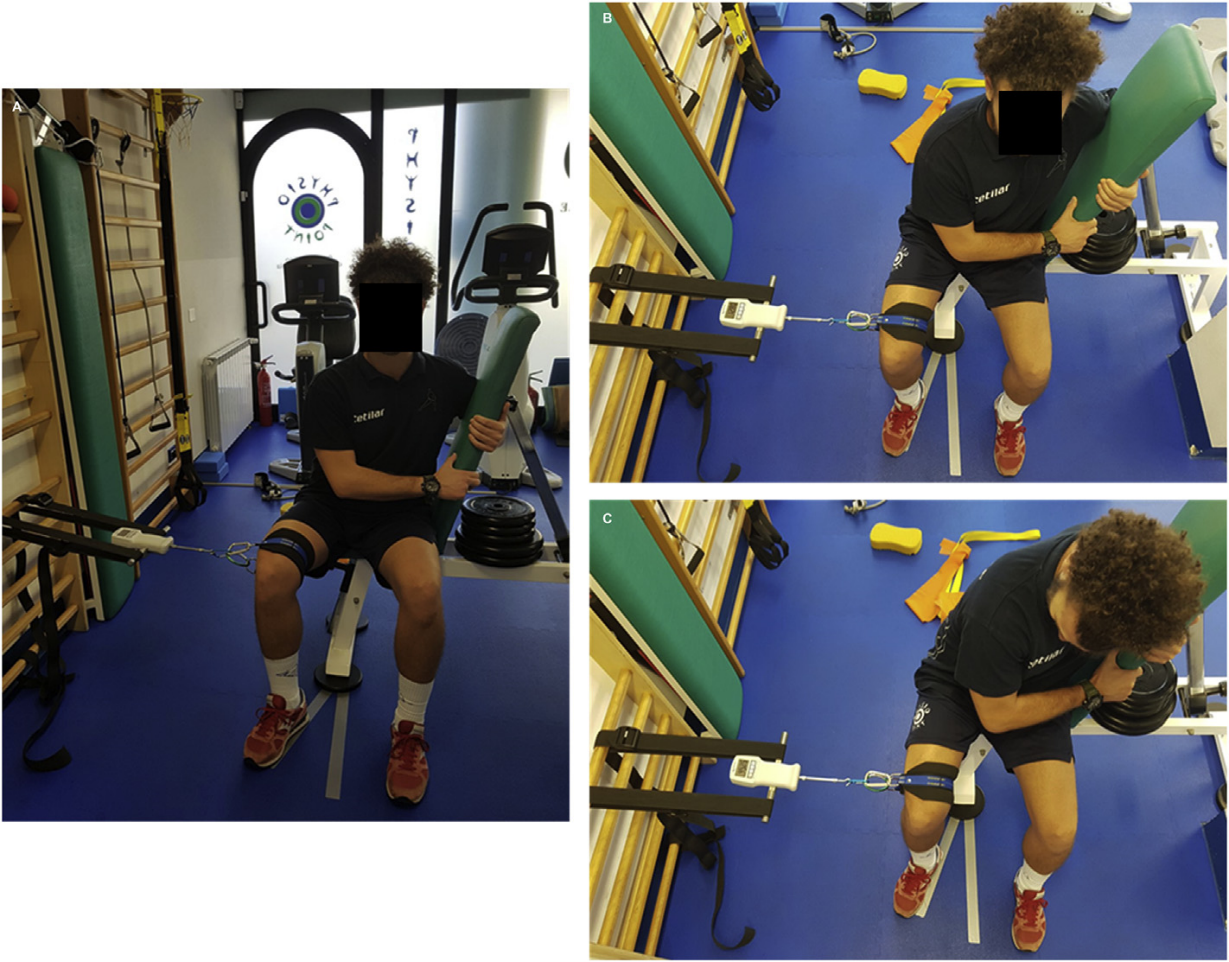
Strength test. The photos represent how the strength test was performed. **Panel A** shows the position of the professional roller hockey player at rest holding the backpad. **Panel B** shows the position of the leg opened at 20°. **Panel C** shows the professional roller hockey player during the test.

The test was performed once a week with the PRHP sitting on an anchored bench in an upright position, with an arm around the backpad and hands clasped in order to increase trunk stability during the test. Hips, knees and ankles were positioned at 90° and limbs opened at 20° in a resting position. The dynamometer was anchored to a headboard and attached to a strap positioned around the limb at one-third distal femur level (Figure 5A, B, and C). The PRHP was then asked to produce a concentric and isotonic contraction of the adductor to his maximum effort. The test was performed on both limbs.

AP-related pain was evaluated using VAS score before the beginning of treatment and every weekday during the entire study period. Traumatic injuries and muscle-tendon functional overloads of the pubic area sustained by the PRHP were also recorded during the study period.

### 5.3 Statistical analyses

Variables were expressed as mean ± standard deviation or count and proportion. Comparisons were performed with unpaired sample *t* test. Response curves were compared using two-way ANOVA with Sidak post-analysis test. Computations were performed in GraphPad Prism 6 (GraphPad Software). A *p* value < 0.05 was considered statistically significant.

## Declarations

### Author contribution statement

E. Pampaloni: Conceived and designed the experiments; Performed the experiments; Contributed reagents, materials, analysis tools or data; interpreted the data.

E. Pera and G. Tarantino: Conceived and designed the experiments; Analyzed and interpreted the data; Wrote the paper.

D. Maggi, R. Lucchinelli, D. Chiappino, A. Costa and V. Venturini: Performed the experiments.

### Funding statement

This research did not receive any specific grant from funding agencies in the public, commercial, or not-for-profit sectors.

### Competing interest statement

The authors declare the following conflict of interests: E. Pera is an employee of Alesco Srl and G. Tarantino is an employee of Pharmanutra SpA.

### Additional information

No additional information is available for this paper.
