# Corrigendum to “Association of cetylated fatty acid treatment with physical therapy improves athletic pubalgia symptoms in professional roller hockey players” [Heliyon Volume 6, Issue 7, July 2020, Article e04526]

**DOI:** 10.1016/j.heliyon.2025.e43329

**Published:** 2025-04-15

**Authors:** Enrico Pampaloni, Elena Pera, Duilio Maggi, Riccardo Lucchinelli, Dante Chiappino, Andrea Costa, Veronica Venturini, Germano Tarantino

**Affiliations:** a Physiotherapy Center, Physio Point, Lucca, Italy; b Alesco Srl, Pisa, Italy; c USL 12, Versilia, Italy; d Diagnostic Imaging OU, Massa Hopsital, Massa, Italy; e Pharmanutra SpA, Pisa, Italy

In the original published version of this article, the authors omitted information on why verbal consent was obtained for participants instead of written consent.

The original statement in section ‘5.1. Study design and participants’ is below:

*In this open-labeled study, 10 PRHPs (Table 1) were enrolled during the A-league season. The PRHPs were not using other topical treatment in the pubic area or oral pain reduction therapy prior to the beginning of the study and did not have any ongoing infectious diseases. PRHPs involved in the study started agonistic activity around the age of 4 years and they all started to have AP at the beginning of their professional activity, around the age of 17 years. Before study start, verbal informed consent was obtained from all participants. In addition, the participant involved in the case study consented to publish his individual details. The study was conducted in accordance with the Declaration of Helsinki and the principles of Good Clinical Practice. Owing to its design and population, the present study did not require approval of an ethics committee*.

This statement has been updated to include information on why verbal consent was obtained instead of written consent.

The correct version of the consent statement can be found below:

***5.1 Study design and participants***

*In this open-labeled study, 10 PRHPs (Table 1) were enrolled during the A-league season. The PRHPs were not using other topical treatment in the pubic area or oral pain reduction therapy prior to the beginning of the study and did not have any ongoing infectious diseases. PRHPs involved in the study started agonistic activity around the age of 4 years and they all started to have AP at the beginning of their professional activity, around the age of 17 years. Before study start, verbal informed consent was obtained from all participants* ***, according to the DIRECTIVE 95/46/EC OF THE EUROPEAN PARLIAMENT AND OF THE COUNCIL of 24 October 1995 on the protection of individuals personal data (Art. 7; https://eur-lex.europa.eu/legal-content/EN/TXT/HTML/?uri=CELEX:31995L0046)*** *In addition, the participant involved in the case study consented to publish his individual details. The study was conducted in accordance with the Declaration of Helsinki and the principles of Good Clinical Practice* ***and was based-on a non-invasive medical device, already CE-marked, used in full compliance with the approved and registered indications for use, with no commercial purposes. Data collection was carried out anonymously and without additional invasive procedures compared to normal clinical practice. For this reason, according with the legislation in force at the time of the study (i.e. D.Lgs. 46/1997 Article 14 and Attached X), the opinion of the local ethics committee was not requested*.**

The ***authors*** apologize for the errors.

## Declaration of competing interest

The authors declare the following conflict of interests: E. Pera is an employee of Alesco Srl and G. Tarantino is an employee of Pharmanutra SpA.

